# Establishment of Callus Cultures from *Dalbergia sissoo* Leaf Explants for Production of Skin Therapeutics: An In Vitro and In Silico Study

**DOI:** 10.3390/molecules30173531

**Published:** 2025-08-29

**Authors:** Jyotsana, Promila Gupta

**Affiliations:** Agriculture Plant Biotechnology Laboratory (ARL-316), University School of Biotechnology, Guru Gobind Singh Indraprastha University, Sector 16-C, Dwarka, New Delhi 110078, India; jyotsana0804@gmail.com

**Keywords:** *Dalbergia sissoo*, callus culture, antioxidant activity, GC-MS, molecular docking, anti-tyrosinase activity, phytochemicals, Molecular Dynamics Simulation

## Abstract

*Dalbergia sissoo* is a commercially exploited timber tree also known for its varied phytochemical constituents holding significant importance in folk medicines with documented biological properties. The present study reports the establishment of callus cultures from its leaf explants for the in vitro production of skin therapeutics. The growth parameters of the callus cultures were calculated. The antioxidant potential of the methanolic extracts of leaf and its callus cultures was evaluated through DPPH assay. Calli at third subculture stage showed the highest antioxidant potential (IC_50_ 273 ± 14.14 µg/mL). A comparative analysis of phytochemical composition was performed using Gas Chromatography–Mass Spectrometry (GC-MS) which revealed the presence of potential skin therapeutic compounds. Out of 146 compounds, only 15 are unique to leaf explants, with the rest being produced in callus cultures. ADME predictions of potential compounds showed their drug likeness properties. The molecular docking of selected phytochemicals such as Chondrillasterol, Stearic acid, and n-Hexadecanoic acid against the tyrosinase enzyme showed better binding affinities than the reference drug (Kojic acid). Molecular dynamics simulation also showed stable conformations of the docked complexes with the target protein. Overall, these investigations unveil for the first time the successful in vitro production of skin therapeutics from *D. sissoo*, ensuring the sustainable and conservation-friendly utilization of its biomass for medicinal purposes.

## 1. Introduction

Since ancient times, plants have been used as a reservoir of traditionally important natural products. They have been used as remedies for various diseases and became an important component for the healthcare system globally [1,2]. The World Health Organization (WHO) emphasizes the importance of plants, as around 80% of the world population depends on herbal products (mostly traditional medicines) for their daily healthcare [3]. In the developing world, the overreliance on chemically synthesized drugs has raised serious concerns such as drug resistance, higher cost, adverse side effects, and ecological footprint. Therefore, the global shift towards the use of natural products has gained attention for treating health conditions as they are known for their safety, efficacy, and broad health benefits with minimum side effects [4].

In an organism, skin is the most extensive and important organ of the body. Skin is subjected to many intrinsic as well as extrinsic stress factors which affect its structure and physiological functions. Extrinsic factors mainly include exposure to UV rays, microbial infections, and chemical agents. These factors cause an imbalance in the number of free radicals or reactive oxygen species which further results in deleterious skin conditions such as photo aging, pigmentation, inflammation, and skin cancer, to name a few [5,6,7]. Free radicals are unstable ion species with unpaired electrons which combine with other molecules to become stabilized and result in oxidative stress. They initiate a chain reaction where free radicals like superoxide radicals, hydroxyl radicals, and peroxyl radicals are produced, causing damage to the skin, ranging from cell damage to cell death. To counteract this process, modern industry has mobilized its extensive research towards natural antioxidants, further highlighting the role of secondary metabolites. They react with free radicals to break the chain reaction ensuring minimum deleterious effects [1,8].

Consequently, among the various deleterious effects of free radicals, the excessive production of melanin is a primary concern which causes numerous skin-related conditions. Melanin is a widely known pigment in nature whose function is to give color to skin, hair, and plant browning [9]. It also plays an important role in the protection from harmful UV rays and oxidative stress [10]. The overproduction of melanin leads to hyperpigmentation, freckles, age spots, post-inflammatory hyperpigmentation, and melasma [11]. Tyrosinase, a copper-containing enzyme, is a key enzyme involved in the melanogenesis pathway which leads to melanin production. The dysregulation of this enzyme results in many disorders. Therefore, the controlled inhibition of this enzyme is crucial for controlling skin-related conditions [12]. Synthetic tyrosinase inhibitors such as kojic acid, arbutin, azelaic acid, and hydroquinone are widely used for their demonstrated efficacy. However, the literature highlights the rising concern over their safety and adverse side effects such as skin irritation and cytotoxicity in association with prolonged use [9,13]. Therefore, researchers have been prompted to identify natural plant-based tyrosinase inhibitors with minimal side effects and maximum efficacy. Many plant-based secondary metabolites (flavonoids, phenolic acids, and tannins) are reported to be used as natural tyrosinase inhibitors [14,15,16]. Secondary metabolites are known as low-molecular-weight compounds which are generally overproduced in plants during stress conditions that perform many important physiological functions such as pollinators/dispersal agent’s attractants, stress tolerance, and pathogen resistance. They are currently used on a large scale in pharmaceutical industries, cosmetics, chemicals, dyes, and as dietary supplements [17].

Consequently, with the increasing demand for plant-derived products in the modern world, skin-related conditions have caused the emergence of ecological issues such as the overexploitation of many medicinal plants, loss of natural habitats, loss of genetic diversity, and even species extinction [18]. Therefore, to circumvent these limitations, conventional biotechnological methods such as plant tissue culture techniques, particularly callus culture, have been reported to be used as a controlled strategy to produce beneficial phytochemicals. This technique highlights the sustainable and continuous production of phytochemicals of interest in an optimized environment by maintaining an ecological footprint [19,20,21]. Callus cultures of *Pelargonium graveolens*, *Centella asiatica*, *Dalbergia ecastaphyllum*, and *Oryza sativa* L. showed comparable results for various activities such as tyrosinase inhibition-enhanced phenolic accumulation, fibroblast protection, anti-aging, strong antioxidant capacity, and UV protection [6,22,23,24].

*Dalbergia sissoo* Roxb., mainly known as Indian rosewood (shisham), belongs to the Fabaceae family and is native to the Indian subcontinent and Southern Iran. It is primarily used for its high-quality timber, as fuelwood, and traditionally for medicinal properties. In ancient and traditional medicine, it is known for its biomodulatory properties which include wound healing, anti-inflammatory, antioxidant, antimicrobial, antidiabetic, antibacterial, neuroprotective, and anti-osteogenic activity [25,26,27]. Ethnopharmacological data has revealed that its parts, mainly the leaf, bark, roots, and wood, were used in treating skin disorders, chronic wounds, inflammatory conditions, and skin infections [28]. The oil has been used to treat ulcers and skin conditions when applied topically. The bark extracts have been used to treat blood disorders, dyspepsia, dysentery, leukoderma, anal disorders, and skin disorders [29]. The wood in powdered form is used as a cooling agent and expectorant for leprosy, scabies, stomach issues, and blood disorders. The leaf extracts showed antipyretic, anti-inflammatory, and antioxidant properties and were used in the treatment of cancer, diabetes, gonorrhea, osteoporosis, and syphilis. The seeds are used for treating burning sensations and scabies [30,31]. Phytochemical investigations have reported that *D. sissoo* exhibits a vast phytoconstituent profile. The leaf extracts showed the presence of flavonoids (biochanin A, genistein, caviunin-7-O-glucoside, and biochanin-7-O-glucoside), phenols, glycosides, and lipids. The stem, bark, and heartwood extracts include compounds such as dalbergin, dalbergichromene, isodalbergin, nordalbergin, tectorigenin, and 4-phenylchromene. The extracts of mature pods are reported to have glycosides like tectoridin and isocaviudin [25,30,31]. In recent decades, due to environmental factors and broad-spectrum properties, the mortality rate of *D. sissoo* has increased, which has raised a serious cause of concern [32].

Despite the presence of the available literature, there exists a gap in the comparative evaluation of the phytochemicals identified in the natural plant-derived extracts and their in vitro raised cultures. Therefore, by using a callus culture technique, the present study was designed to assess the comparative phytochemical characterization using a Gas Chromatography–Mass Spectrometry (GC-MS) analysis of both a leaf extract and its callus culture extract at different subculture stages. Further in vitro biochemical assays were performed for evaluating antioxidant potential and an in silico assessment of tyrosinase inhibition potential was performed, as they are key indicators of cosmeceutical importance.

## 2. Results and Discussion

Based on the previous literature reported, plant tissue culture techniques were utilized for the micropropagation and conservation of the *Dalbergia sissoo* [33]. Therefore, the present study reports, for the first time, a comparative analysis of the phytochemicals identified from leaf explant (LE) and its callus cultures at different subculture stages for the production of secondary metabolites with skin therapeutic potential. Furthermore, the antioxidant potential was also evaluated for all the extracts. Additionally, the potential phytochemicals were selected based on their skin therapeutic potential and further studied through in silico analysis for their inhibition activity against the tyrosinase enzyme.

### 2.1. Callus Culture Initiation

**Figure 1:**
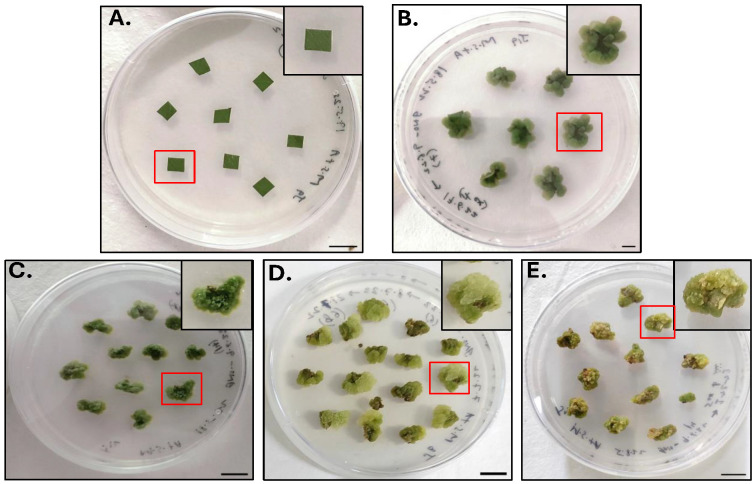
Pictorial representation of Petri plates showing (**A**) inoculation of leaf explant (LE), (**B**) callus induction till 20th Day (20DC), (**C**) 1st subculture stage (20DC1), (**D**) 2nd subculture stage (20DC2), (**E**) 3rd subculture stage (20DC3). Inset: close-up view highlighting the single explant and individual callus. Scale bar: 1 cm.

Previous studies suggested that callus induction and proliferation depend upon the type of hormone treatment, type of medium, and concentration of plant growth regulators (mainly auxin and cytokinin) [34,35]. In the present data, primary callus was initiated from the LE placed on the Murashige and Skoog (MS) medium supplemented with the growth regulators BAP (0.5 mg/L) and NAA (3.5 mg/L) (Figure 1A). This agrees with the data which reported that auxin and cytokinin were important for the dedifferentiation of the explant for callus induction [36].

In the present study, callus initiation started after 7 days of inoculation when the explant started becoming swollen from the wounded site followed by curling. The callus emerged from the wounded site first, then from the whole explant (Figure 1B), confirming the effect of both endogenous and exogenous plant growth regulators [36].

After 20 days of callus formation, Fresh weight (FW) and Dry weight (DW) were found to be 33.88 ± 2.74 g and 4.43 ± 0.14 g, respectively. The moisture content (MC) and yield of the callus cultures were found to be 86.83 ± 0.76% and 13.16 ± 0.76%, respectively. This data revealed that the callus produced by continuous cell division resulted in a high biomass accumulation and water content in the presence of growth hormones. The callus induction frequency (CIF) was evaluated to be 86.24 ± 7.32% which indicates that leaf explant is more suitable for callus induction, which agrees with earlier reports [35].

The callus morphology (color and texture) was observed at each stage of callus induction. Callus color and texture demonstrates the quality of the callus [37]. The primary calli (20DC) (Figure 1B) were green in color, which indicates active cell division and the production of pigments, compared with 1st subculture stage (20DC1) (Figure 1C), 2nd subculture stage (20DC2) (Figure 1D), and 3rd subculture stage (20DC3) (Figure 1E) calli which were light green, whitish green, and brownish green in color, respectively. This observation agrees with the literature which reported the changes in color with every subculture stage which indicates the accumulation of more phenolic compounds and sugars [38].

The texture analysis of calli showed that 20DC showed a more compact callus compared with the calli at the subculture stages which showed a less compact and slightly friable callus. The texture changes depend on the response of cells towards the amount of growth hormones in medium [38]. The callus texture observed in the primary stage and after subculture stages were found to be in accordance with the results of earlier studies which reported the effect of growth regulators on callus morphology [39,40]. Thus, the results obtained showed that the callus becomes successfully induced from the leaf explant and proliferated on similar medium composition at different subculture stages.

### 2.2. Antioxidant Potential Assay

The antioxidant potential of all the methanolic extracts was determined by evaluating their potential to scavenge DPPH. DPPH is known as a free radical which becomes reduced in the presence of a hydrogen-donating antioxidant compound. DPPH gives a purple color in free form and after being reduced by the hydrogen donor, the decolorization is measured quantitatively by a decrease in absorbance at 517 nm [41]. Ascorbic acid (AsA) was used as a standard reference compound.

The results showed that the methanolic extract of 20DC3 has a maximum antioxidant potential with an IC_50_ value of 273 ± 14.14 µg/mL which is lower than the IC_50_ values of other methanolic extracts. 20DC3 showed higher antioxidant activity in comparison with parent leaf explant mainly due to the in vitro conditions which may be attributed to the upregulation of certain metabolic pathways such as the phenylpropanoid pathway, resulting in the production of several phenolic compounds while ensuring higher antioxidant activity. Likewise, this agrees with the results of the higher antioxidant potential of callus extract derived from *Centella asiatica* compared with their parent plant [6]. Furthermore, the data correlates with the findings of Mustafina et al. [42] showing that callus extracts have a higher antioxidant potential with a lower IC_50_ value than the crude plant extract. Moreover, previous research revealed that methanolic extraction leads to the maximum extraction of compounds such as phenolic compounds, fatty acids, and sugars which further contributes to the higher antioxidant activity of 20DC3 [43]. However, the least antioxidant potential was shown by 20DC2 with an IC_50_ value of 749 ± 18.66 µg/mL which is comparable with 20DC1 having an IC_50_ value of 726 ± 6.20 µg/mL. The comparative IC_50_ values for all the extracts are shown in Table S1.

**Figure 2:**
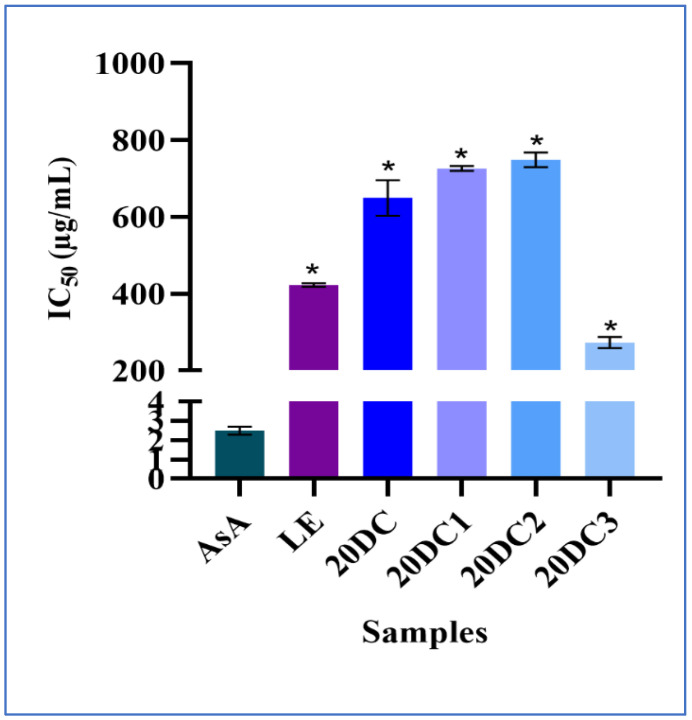
Comparison of 50% inhibition of DPPH free radicals by methanolic extracts of LE, 20DC, 20DC1, 20DC2, and 20DC3. Values are reported as Mean ± SD (*n* = 3). * Values are statistically significant showing *p* < 0.0001.

The trend in the antioxidant potential of all the extracts in decreasing order was 20DC3 > LE > 20DC > 20DC1 > 20DC2 (Figure 2). This may be due to various reasons such as difference in harvest time, light conditions, and extraction process [44]. However, the data presented proves to be statistically significant but future investigations are warranted for assessing the higher antioxidant activity of 20DC3 for its potential applications.

### 2.3. Analysis of GC-MS Chromatograms 

The identification and characterization of the phytochemicals present in the methanolic extracts of all the samples was performed using GC-MS analysis. After an experimental run of 40 min, many peaks were observed in the chromatograms which confirmed that the samples are rich in bioactive compounds (Figure S1). Each peak corresponded to a phytochemical which was identified by relating the retention time (RT), molecular weight (MW), molecular formula (MF), and mass spectrum of the known compounds indexed in the NIST 14s library and the WILEY 08 library [45]. The chromatograms revealed that a total of 146 phytochemicals were present in all the samples, out of which the maximum number of compounds was 36 in 20DC. The trend in decreasing order was 36 (20DC) > 31 (20DC2) > 30 (20DC1) > 25 (20DC3) > 24 (LE). This trend highlights that the number of phytochemicals detected in callus culture extracts was more in comparison to leaf extracts. This result correlates with the studies which previously reported that the number of secondary metabolites produced in a callus is affected by the growth conditions, environment, and growth regulators present during callus induction which acts as a stressor for phytochemical production [38]. Overall, the chromatograms showed the presence of a diverse class of metabolites such as terpenoids, fatty acids and their esters, alkanes, alcohols, and sterols possessing promising biological activities as reported in the previous literature for *D. sissoo* [25,26,27,28,46].

**Figure 3:**
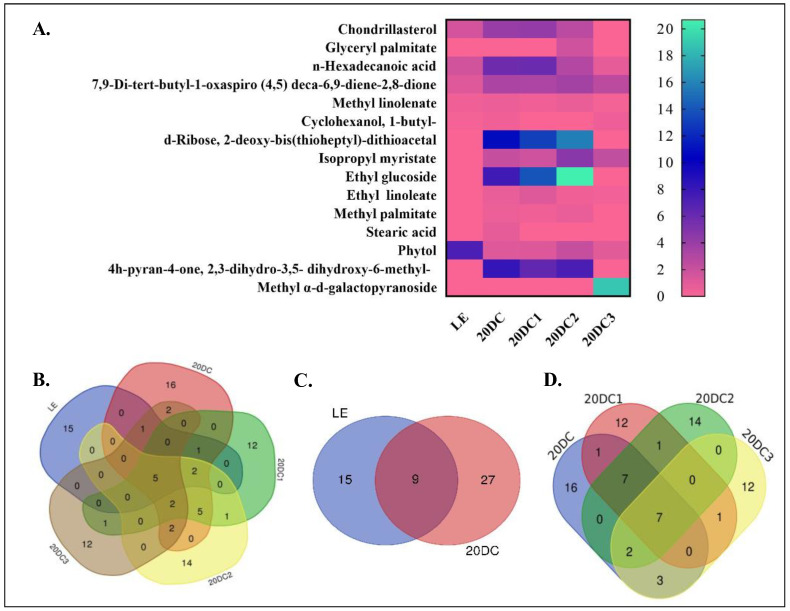
(**A**) Heat map analysis of shortlisted compounds based on their comparative peak percentage area. The Venn diagram represents the common compounds present in (**B**) LE, 20DC, 20DC1, 20DC2, and 20DC3; (**C**) LE and 20DC; (**D**) 20DC, 20DC1, 20DC2, and 20DC3.

For the identification of common compounds in all the samples, a Venn diagram was generated using a freely available tool (https://bioinformatics.psb.ugent.be/webtools/Venn/) (accessed on 3 June 2025). Additionally, a heat map was also generated for assessing the comparative peak area of major abundant compounds (Figure 3A). Among 146 compounds detected, only 5 compounds (7,9-Di-tert-butyl-1-oxaspiro (4,5) deca-6,9-diene-2,8-dione, Phytol, 1,2-Benzenedicarboxylic acid, n-Hexadecanoic acid, and Methyl linolenate) were common in the leaf explant and all callus cultures, highlighting the novelty of in vitro-produced compounds (Figure 3B). These compounds might have been produced in the leaf and continued to be produced at every culture stage. The Venn diagram of the leaf explant and primary callus, i.e., 20DC, showed that 9 compounds are common in both while 27 compounds were unique and produced in the callus only (Figure 3C). Furthermore, if we compare the compounds identified in all callus cultures, their Venn diagram depicts that seven compounds were common with a different peak area percentage in each sample (Figure 3D).

The present study highlights the comparative abundance of certain compounds at one or another stage of culture/subculture. The production of some compounds such as 7,9-di-tert-butyl-1-oxaspiro(4,5)deca-6,9-diene-2,8-dione, phenol,2,4-bis(1,1-dimethylethyl)-, chondrillasterol, 1,2-benzenedicarboxylic acid, n-hexadecanoic acid, and methyl linolenate increased in the callus culture extracts whereas phytol production decreased when compared with the leaf extract. Based on the literature, the most relevant compounds detected in the methanolic extracts, with their MF, MW, comparative peak percentage area, and biological activities, are listed in Table 1.

The methanolic extract of leaf explant revealed the presence of 24 compounds among which the major compounds were mome inositol (35.35%); quinic acid (20.11%); phenol, 2,4-bis(1,1-dimethylethyl)- (10.18%); lupeol (7.39%); phytol (7.22%); biochanin a (4.93%); n-hexadecanoic acid (1.67%); chondrillasterol (1.57%); and squalene (1.59%). Furthermore, the GC⁃MS chromatogram of 20DC displayed the maximum number of peaks which correspond to compounds such as phenol, 2,4-bis(1,1-dimethylethyl)- (39.03%); n-hexadecanoic acid (5.75%); chondrillasterol (4.00%); 4h-pyran-4-one,2,3-dihydro-3,5-dihydroxy-6-methyl- (DDMP) (8.15%); ethyl glucoside (7.60%); isopropyl myristate (2.15%); phytol (1.09%); neophytadiene (1.05%); and stearic acid (0.82%).

Similarly, in the methanolic extract of 20DC1, the abundance of ethyl glucoside (13.94%); d-ribose,2-deoxy-bis(thioheptyl)-dithioacetal (13.08%); phenol, 2,4-bis(1,1-dimethylethyl)- (27.04%); n-hexadecanoic acid (5.86%); phytol (1.14%); DDMP (6.23%); chondrillasterol (4.16%); and ethyl linoleate (1.11%) was detected. Moreover the GC⁃MS profiling of 20DC2 revealed about 31 compounds, among which the maximum peak area was covered by compounds such as phenol, 2,4-bis(1,1-dimethylethyl)- (21.93%); ethyl glucoside (20.67%); ddmp (7.19%); d-ribose,2-deoxy-bis(thioheptyl)-dithioacetal (15.74%), isopropyl myristate (4.64%); phytol (2.13%); chondrillasterol (2.57%); and n-hexadecanoic acid (2.90%). On the contrary, the methanolic extract of 20DC3 showed a lower number of compounds compared with the other subculture stages and the major composition included phytochemicals such as phenol, 3,5-bis(1,1-dimethylethyl)- (58.34%); methyl α-d-galactopyranoside (MGP) (18.83%); 1,5-anhydro-6-deoxyhexo-2,3-diulose (3.43%); 1,2-benzenedicarboxylic acid (2.95%); 7,9-di-tert-butyl-1-oxaspiro (4,5)deca-6,9-diene-2,8-dione (2.53%); isopropyl myristate (2.26%); phytol (0.92%); and n-hexadecanoic acid (0.77%).

Based on the comparative analysis of the phytochemical composition of all the methanolic extracts shown in the present study, the results implicated the therapeutic importance of the plant and its callus cultures, as these compounds are reported in the literature for their broad-spectrum activities. Phytol, a diterpene, present in all the methanolic extracts, is known to have antioxidant, antimicrobial, anti-inflammatory, anticancer, anti-melanogenesis, and anti-acne properties with a presence in many plants [51,58,67,68]. Stearic acid, a saturated fatty acid detected in 20DC only, shows anticancer, antioxidant, antimicrobial, and antibacterial properties [51,52]. It also exhibits skin therapeutic potential, due to its moisturizing and smoothening effect, as an anti-aging agent and acts as an emollient when present in topical formulations [69,70,71]. n-Hexadecanoic acid, commonly known as palmitic acid, is also a saturated fatty acid found in all the methanolic extracts, showing antioxidant, anti-inflammatory, nematicide, hypocholesterolemic, and anti-androgenic properties [59,61]. Its multiple skin therapeutic potential is reported in previous studies as an emollient, surfactant, and moisturizing agent and it can act as a skin barrier and have an anti-inflammatory effect [60,72]. Likewise, hexadecanoic acid methyl ester or methyl palmitate, present in maximum amounts in 20DC2, is a saturated fatty acid which is reported to show anti-inflammatory, anti-androgenic, immunostimulant, chemoprotective, antitumor, and antimicrobial activities [51,56,57,58]. It is primarily used in skincare for its emollient, anti-inflammatory, skin smoothening, and skin conditioning properties [72,73].

The compound methyl linolenate is a methyl ester of linolenic acid and found in maximum quantity in 20DC2. It exhibits antioxidant, anticancer, anti-melanogenesis, and anti-inflammatory properties. In the literature, it shows promising skin therapeutic potential, particularly for its skin whitening and emollient properties [64]. Similarly, another derivative of linolenic acid, ethyl linoleate, is widely known for its anti-inflammatory, antioxidant, anti-melanogenesis, and antimicrobial properties. In the context of skin therapeutics, it is being explored for its anti-acne potential, skin brightening effects, and function as an emollient [65,66].

Another important compound, isopropyl myristate, formed by the esterification of isopropanol and myristic acid, was found to be present in all the calli extracts. It is used widely in cosmetics for its emollient effects, skin conditioning properties, as an emulsifier and binding agent, and its antioxidant and antibacterial activity [51,52]. Moreover, Phenol, 3,5-bis(1,1-Dimethylethyl)-, which is the most abundant compound detected in 20DC3, has been reported to have antifungal, antioxidant, antimicrobial, and antidiabetic properties [56]. Therefore, it can be highlighted that the abundance (58%) and antioxidant property of this compound might be the reason for higher antioxidant potential in the 20DC3 extract.

Thus, an overall comprehensive analysis of the phytochemical profiling of all the callus culture stages showed the presence of numerous skin therapeutic phytoconstituents reported in the literature at more than one stage such as phytol, methyl linolenate, ethyl linoleate, isopropyl myristate, n-hexadecanoic acid, methyl palmitate, stearic acid, glyceryl palmitate, and ethyl glucoside. However, apart from these major compounds, there is the presence of some important phytochemicals having a peak area percentage of less than 1% present individually in the samples, possessing important biological properties. It can be elucidated from the present study that callus cultures can prove to be an efficient method for the production of these important skin therapeutic phytochemicals.

Although the present study emphasizes the preliminary exploration of callus cultures over longer durations with continuous subculturing for phytochemical profiling, it also highlights the limitations for time efficiency and scalability for future applications. Therefore, future investigations regarding callus culture duration, elicitor treatment, and growth conditions could be focused on the enhanced production of phytochemicals from selected callus stage within shorter durations. Additionally, further studies such as bioprospecting should be explored for their implicated biological properties.

### 2.4. In Silico Analysis

In silico analyses are gaining interest these days as studies focused on the identification of phytochemicals from natural plants are favoring attention from the pharmaceutical industry over synthetic products. Bioinformatics tools enable us to identify the potential compounds and assess the interaction between the tested ligands and target protein [74,75]. In the present study, the selected ligands from the GC-MS analysis of methanolic extracts were tested for their anti-tyrosinase activity against the mushroom tyrosinase target protein.

#### 2.4.1. ADME Predictions

The compounds which follow Lipinski’s rule of five are considered as potential drug candidates. Lipinski’s rule states certain parameters which are as follows: the molecular weight (MW) < 500, number of hydrogen bond donors (HBD) < 5, number of hydrogen bond acceptors (HBA) < 10, and the octanol–water partition coefficient (M Log *P*, lipophilicity index) ≤ 5 [13,76]. Furthermore, other parameters such as the number of rotatable bonds and skin permeation coefficient (Log *Kp*) were also reported using software. The compounds with favorable parameters and minimum violation of the rules (≤1) were selected subsequently for further studies and listed in Table 2. The compounds Chondrillasterol, Stearic acid, Methyl palmitate, Isopropyl myristate, Phytol, and n-Hexadecanoic acid showed one violation by exceeding the threshold value of Log *P*, while the rest of the other compounds showed no violation of Lipinski’s rule.

The skin permeation coefficient relates to the ability of certain compounds to pass through membranes. A lower value of Log *Kp* for a particular compound suggests the lesser probability of its skin permeation [77]. Ethyl glucoside and MGP exceeded the threshold value of Log *Kp* with values of −9.19 cm/s and −9.37 cm/s, which revealed that they are less permeable through skin. The skin permeation coefficient order for all of the compounds were reported as follows: Stearic acid > Phytol > Methyl palmitate > n-Hexadecanoic acid > Isopropyl myristate > Chondrillasterol > Glyceryl palmitate > D-Ribose, 2-deoxy-bis(thioheptyl)-dithioacetal > 1-Butyl-cyclohexanol > 7,9-Di-tert-butyl-1-oxaspiro (4,5) deca-6,9-diene-2,8-dione > Ethyl linoleate > Methyl linolenate > DDMP > Ethyl glucoside > MGP. The present study showed that the selected compounds successfully followed Lipinski’s rule with no or a minimum of one violation and have acceptable pharmacokinetic properties. Based on the literature related to their skin therapeutic potential, out of these selected compounds, only 12 compounds were further evaluated for their anti-tyrosinase property.

#### 2.4.2. Molecular Docking Analysis

Molecular docking analysis is a widely used technique in drug development, which examines the interaction between the ligands and the target protein by identifying the amino acid residues involved during the interaction [12].

The tyrosinase enzyme is reported to be involved in melanin production, which results in skin pigmentation. The dysregulated function of the enzyme results in various skin disorders, therefore its inhibition is targeted and studied widely for the treatment of hyperpigmentation, freckles, dark spots, and melasma [10,12]. Its structure comprises a tetramer unit, in which active sites containing two copper ions are present at the catalytic site. These ions mainly coordinate with histidine amino acid residues (His85, His61, His94, His263, His259, and His296). Furthermore, other amino acid residues are also included in the interaction with native tropolones, which are Phe264 and Val283. Previous studies reported that the selected ligands must actively interact with these amino acid residues for the inhibition of the tyrosinase enzyme [12,78,79]. To identify the binding affinity, molecular docking was performed using Auto Dock Vina using the crystallographic structure of the target protein tyrosinase with PDB ID: 2Y9X with a native inhibitor tropolone [80].

**Figure 4:**
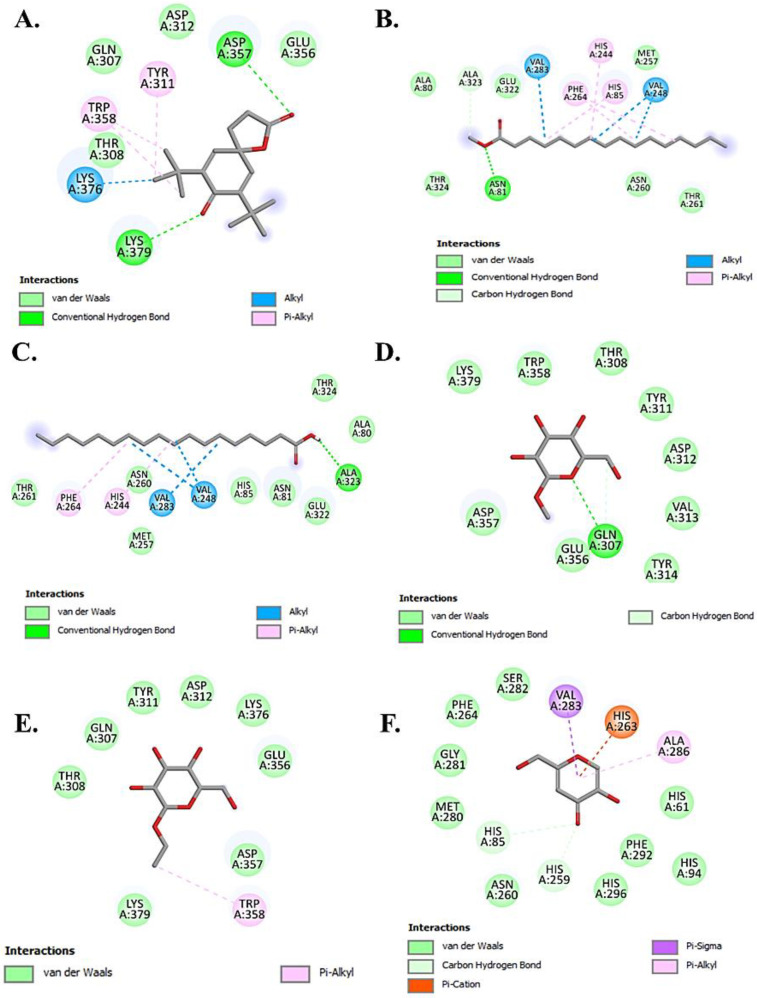
Representation of 2D molecular interactions between the target protein 2Y9X and the tested ligands (**A**) 7,9-Di-tert-butyl-1-oxaspiro(4,5)deca-6,9-diene-2,8-dione, (**B**) Methyl palmitate, (**C**) Stearic acid, (**D**) Methyl α-d-galactopyranoside, (**E**) Ethyl glucoside, and (**F**) Kojic acid.

**Figure 5:**
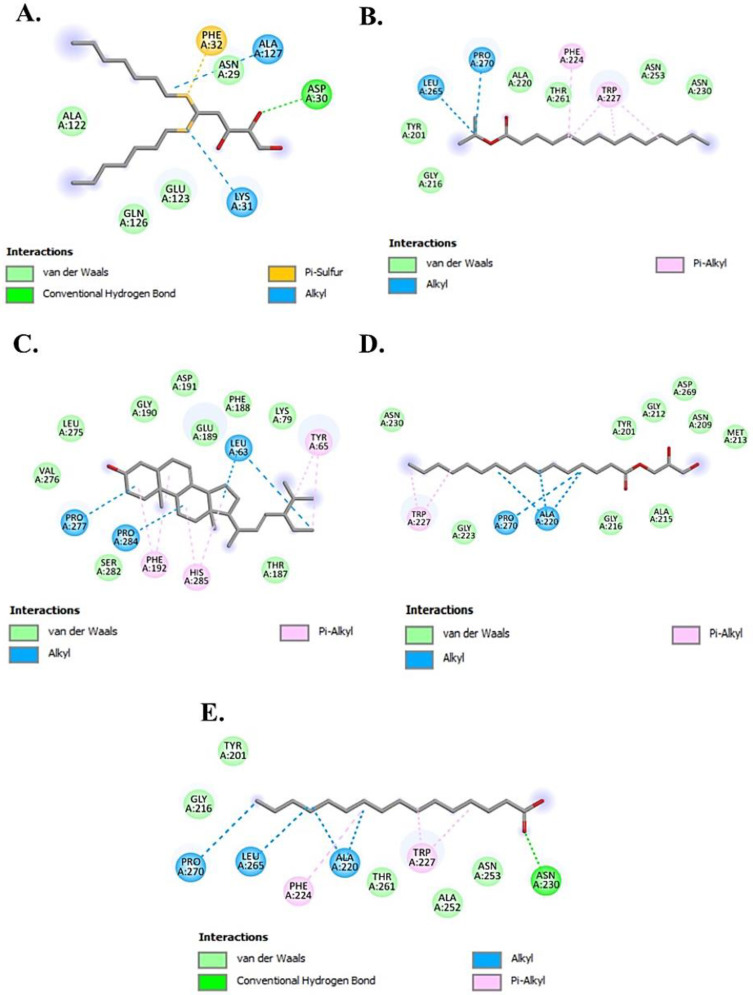
2D molecular interactions between the tested ligands and target protein 2Y9X showing (**A**) D-Ribose, 2-deoxy-bis(thioheptyl)-dithioacetal, (**B**) Isopropyl myristate, (**C**) Chondrillasterol, (**D**) Glyceryl palmitate, and (**E**) n-Hexadecanoic acid.

The molecular docking studies revealed that the selected ligands showed a better binding affinity than the reference inhibitor kojic acid taken as standard (Table 3). The results indicated that Chondrillasterol has shown the strongest binding affinity (−8.7 kcal/mol) which is contributed by Vander Waals and hydrophobic interactions compared with the standard inhibitor kojic acid (−5.9 kcal/mol). The 2D interactions of all the tested ligands are shown in Figure 4 and Figure 5. The results of the molecular interaction of stearic acid with the target enzyme depict that the amino acid residues involved in the interaction are Ala323 through hydrogen bonding; Thr261, Asn260, Met257, His 85, Asn81, Glu322, Thr324, and Ala80 through Vander Waals interaction; and Phe264, His244, and Val283 by hydrophobic interaction at the active site of enzyme. Furthermore, the compound methyl palmitate also showed the interaction with the active sites of the target protein with the involvement of amino acids such as Asn81 and Ala323 through hydrogen bonding and Val283, Phe264, His244, and His85 through hydrophobic interactions. The docking results with other tested ligands also showed a better binding affinity than kojic acid by interacting with other domains of the active site with different amino acid residues, as shown in Table 3.

Therefore, the molecular docking results suggest that potential compounds are present in the methanolic extracts of leaf and its callus cultures, which could possibly have a role in the efficacious inhibition of the tyrosinase enzyme.

### 2.5. Molecular Dynamics Simulation Studies

**Figure 6:**
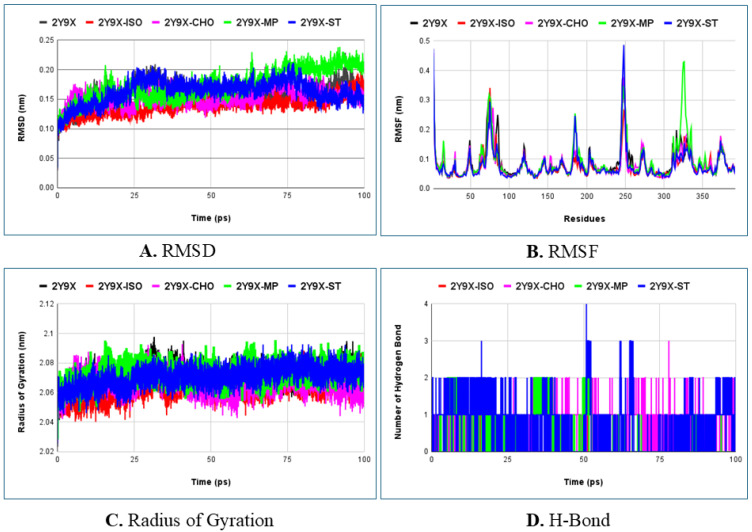
Graphical representation of molecular dynamics simulation depicting (**A**) RMSD, (**B**) RMSF, (**C**) Radius of Gyration, and (**D**) H-Bond parameters of best tested ligands, Isopropyl myristate (ISO), Chondrillasterol (CHO), Methyl palmitate (MP), and Stearic acid (ST) with target protein (2Y9X).

Molecular dynamics simulation was investigated for over 100 ns to fully understand the stability, flexibility, and molecular behavior of the protein–ligand complex interactions at the atomic level. These interactions were evaluated using various parameters such as Root Mean Square Deviation (RMSD), Root Mean Square Fluctuations (RMSFs), Radius of Gyration (Rg), and hydrogen bonding (H-Bond) which are calculated using a time-dependent molecular system [81]. The average values of these parameters (mainly RMSD and Rg) for all tested ligands with the target protein are listed in Table 4. Furthermore, based on the literature, the best docking interactions, skin permeation ability, comparable values of RMSD, RMSF and Rg, the best tested ligands are presented in the graphical plots depicted in Figure 6.

The RMSD values correspond to the stability and equilibrium of the interacting complexes being investigated during the simulation. An overview of the predicted average values of RMSD for all the tested ligands demonstrated that the complexes were stable and well equilibrated throughout the analysis (Table 4). The graph plot illustrated that the ligand–protein complex showed less variation which reveals the greater stability of the system while interacting with the active site of the enzyme. Among the ligands tested, 2Y9X-isopropyl myristate showed the least deviation in comparison to the other complexes, which confirms its stability with a value below 3 Å (Figure 5A). Additionally, the 2Y9X-chondrillasterol and 2Y9X-stearic acid complexes also showed less deviation which also confirms the stability of the system. Conversely, methyl palmitate showed minor deviation at around 75–100 ns with a higher RMSD value which confirms its lesser stability compared with other ligand complexes shown in the graph.

RMSF is an important structural parameter which provides information about the flexibility of the individual residues and fluctuations in the different regions of protein, in response to the ligand binding [79,81]. Lower values of fluctuations signify the stability of the complex. The average RMSF values of all the ligand–protein complexes examined depicted less fluctuations when compared with the reference complex revealing the stability of the systems. The graph demonstrated that complex 2Y9X-Isopropyl myristate and 2Y9X-Chondrillasterol showed lesser fluctuations with values less than 4 Å comparable to the protein and reference ligand–protein complex (Figure 5B). Subsequently the protein complex with Methyl palmitate and Stearic acid showed average values of fluctuations above 4 Å at residue numbers 248 and 326, respectively, which signifies their lower stability within the binding site of the complex.

The Rg is a vital parameter which evaluates the compactness of the protein and protein–ligand complex. It also assesses the stable folding and unfolding nature of the complex in the presence of the interacting ligand. The higher the value of Rg, the lower the compactness (more unfolding) of the complex [82]. The graphical plot of Rg demonstrated that the average value of the protein and protein–ligand complex lies in the range of 20.4–20.9 Å (Figure 5D). Notably, the average value of all protein–ligand complexes assessed in the present study showed the same comparable average value of Rg which depicts the maintenance of compactness and the stable folding of the complexes during the entire process.

The H-Bond analysis exhibits a special role in sustaining the stability of the protein–ligand complexes. Therefore, we analyzed the formation of hydrogen bonds during the 100 ns simulation. The hydrogen bond chart displayed that the number of hydrogen bonds varied in the range of 0–4 in the case of reference complex (2Y9X-kojic acid) which is comparable to the stearic acid complex. Furthermore, the other tested ligands presented in the chart such as isopropyl myristate, methyl palmitate, and chondrillasterol also displayed a number of hydrogen bonds within a similar range. Overall, it can be concluded that the selected ligands exhibited comparable interactions relative to the reference complex.

In the present investigation, although the molecular dynamics simulation provides valuable insights at the atomic level of ligand and protein interactions, there are several limitations when compared with the complexity of a real biological system. Hence this investigation further warrants the in vitro experimental validation depicting the inhibiting role of the tested ligands with the target enzyme.

## 3. Materials and Methods

### 3.1. Chemicals and Reagents

All the chemicals used for callus culture establishment and assays were of analytical grade. For MS medium preparation, potassium dihydrogen phosphate, calcium chloride dihydrate, boric acid, manganese-monohydrate, zinc sulfate heptahydrate, potassium iodide, sodium molybdate di-hydrate, copper sulfate pentahydrate, cobalt chloride hexahydrate, sodium EDTA dihydrate, ferrous sulfate heptahydrate, inositol, glycine, nicotinic acid, sucrose, and pyridoxine hydrochloride were purchased from Sisco Research Laboratories Pvt. Ltd. (SRL), Mumbai, India. Ammonium nitrate, potassium nitrate, and magnesium sulfate heptahydrate were purchased from Merck KGaA, Darmstadt, Germany, Central Drug House Pvt. Ltd. (CDH), Delhi, India, and Thomas Baker (Chemicals) Pvt. Ltd., Mumbai, India, respectively. Gelling agent agar, growth regulators cytokinin (6-Benzyl aminopurine) and auxin (1-Naphthaleneacetic Acid), and thiamine hydrochloride were purchased from Himedia Co., Mumbai, India. For biochemical assays, methanol (HPLC-grade) and dimethyl sulfoxide (DMSO) were purchased from SRL, Mumbai, India. Standards, 2,2-diphenyl-2-picrylhydrazyl (DPPH) and L-ascorbic acid (AsA), were purchased from Sigma Aldrich Co., St. Louis, MO, USA.

### 3.2. Media Preparation

For callus culture initiation, MS medium [83] was prepared using stock solutions along with 0.8% agar and 3% sucrose. The MS medium was supplemented with plant growth regulators auxin (NAA, 3.5 mg/L) and cytokinin (BAP, 0.5 mg/L) using the protocol reported by Ramamurthy and Savithramma with slight modifications [84]. Furthermore, the pH of the medium was adjusted to a range of 5.6–5.8 and autoclaved at 121 °C for 15 min, followed by pouring into Petri dishes.

### 3.3. Establishment of Callus Culture

#### 3.3.1. Plant Collection and Inoculation

*Dalbergia sissoo* leaves were collected from the university premises of Guru Gobind Singh Indraprastha University, Dwarka, Delhi, India. The plant specimen was verified by botanist Dr. Promila Gupta, Professor, University School of Biotechnology (USBT), Guru Gobind Singh Indraprastha University (GGSIPU) and a voucher specimen USBT-JY/1/18 was submitted to the herbarium collection of Agricultural Research Laboratory-316, USBT, GGSIPU.

Fresh and young leaves were selected as the explant and washed with 1% teepol for 10 min followed by washing thrice with distilled water. To minimize the growth of bacterial or fungal infections, a surface sterilization protocol was followed in which leaves were first rinsed with 0.1% mercuric chloride for 1 min in the biological safety cabinet (Haier, Qingdao, China) followed by rinsing thrice with autoclaved distilled water. After removing the excess water, the midrib of the leaf was removed, cut into square segments (5 mm × 5 mm approx.), and placed on the media plates. All the plates with explants were placed under white light with a photoperiod of 16 h light and 8 h dark conditions, at a temperature of 25 ± 2 °C, for a time span of 20 Days. The parameters FW, DW, CIF, yield, MC, and morphology (color and texture) of the callus were recorded after 20 days of callus induction [40,85]. CIF, MC, and yield of the callus were assessed using the following formula:

CIF = (Number of explants induced with callus/Total number of explants cultured) × 100(1)

MC = [(FW of callus − DW of callus)/FW of callus] × 100 (2)

Yield% = (DW of Callus/FW of callus) × 100(3)

#### 3.3.2. Subculture Protocol

After the inoculation of explants on the media plates, the calli were induced from the excised part of the parent explant. These calli were further subcultured by removing the remaining parent explant after every 20 days on fresh media plates till the third subculture stage.

### 3.4. Extract Preparation

Calli collected from primary culture stage (20DC), and from subsequent subculture stages (20DC1, 20DC2, and 20DC3) along with the LE were used for extraction. For the extraction process, 1 g of each sample was weighed and ground into a fine powder using liquid nitrogen. The samples were then mixed with 10 mL HPLC-grade methanol in a 1:10 (*w*/*v*) ratio. The ultrasonication procedure was performed using the Ultrasound Sonicator System (Biologicals Model-3000 MP ultrasonic homogenizer, Manassas, VA, USA) with 4 mm stepped probe at room temperature with 30 s on and off cycles for 1 h and at 40% amplitude [86]. The sample was placed on the ice bath to maintain temperature during ultrasonication. The extracts were then centrifuged at 10,000 rpm for 10 min and filtered using Whatman filter paper 1 (Axiva Sichem Biotech, Sonepat, Haryana, India). Supernatants were collected, oven-dried at 35 °C, and stored at 4 °C till further analysis.

### 3.5. Antioxidant Assay

Stock solutions of the extracts were prepared for performing a DPPH free radical scavenging assay [87]. For the assay, 10 mg/mL stock solution of each extract was prepared using DMSO solvent. Next, 100 µL of each extract was mixed with 900 µL of 0.1 M DPPH (prepared in methanol). The reaction mixture was incubated in the dark at room temperature for 30 min. Furthermore, the absorbance was taken at 517 nm in a spectrophotometer (Jenway, Genova Bio/Cole-Parmer SP-400-BIO UV/Visible Diode Array Scanning Spectrophotometer; 100–240 VAC, 50/60 Hz, Cole Parmer Instrument Company, LLC, Mumbai, India). AsA was used as the positive control. Methanol was used as the blank. The following formula was used to calculate free radical scavenging activity:

50% Inhibition Concentration (IC_50_) = [(A_C_ − A_S_)/A_C_] × 100 (4)

where A_C_ is the absorbance of control and A_S_ is the absorbance of sample.

### 3.6. GC-MS Analysis

The phytochemical composition of the methanolic extracts was analyzed using GC-MS. The analysis was performed on Model GC⁃MS-QP2010 Ultra (Shimadzu Corp., Nakagyo-ku, Kyoto, Japan) at the Advanced Instrumentation Research Facility, Jawaharlal Nehru University, New Delhi, India. The column used was capillary column Rtx-5MS with dimensions 30 m × 0.25 mm × 0.25 µm. Then, 15 mg/mL stock solutions of methanolic extracts of 20DC, 20DC1, 20DC2, 20DC3, and LE were prepared using methanol and filtered through syringe filters (0.2 µm pore size, 25 mm diameter) to remove particles from solution.

The oven temperature was kept at 80 °C initially for 3 min and further increased to 300 °C at a rate of 10 °C/min with a hold time of about 17 min. The injection port temperature was 260 °C and the samples were injected in split mode at a ratio of 10:1. Helium was used as the carrier gas with a flow rate of 1.21 mL/min. The ion source temperature was set to 220 °C with an interface temperature of 270 °C and a mass scan range of 40–650 *m*/*z*. The duration of the GC-MS analysis program was about 40 min [45]. The compounds were identified by comparing the peaks of mass spectra with the data available in the NIST 14s library and WILEY 8 library database. The amount of each identified compound was evaluated using the area under each peak in the chromatogram.

### 3.7. In Silico Analysis of Shortlisted Compounds

Based on the reported literature, the compounds (ligands) with promising biological activities were shortlisted for an evaluation of their skin therapeutic potential using in silico tools.

#### 3.7.1. Drug Likeness (ADME Analysis)

The SwissADME tool (http://www.swissadme.ch/) (accessed on 24 April 2025) was used to study the pharmacokinetic properties (absorption, distribution, metabolism, and excretion), physicochemical properties, and drug likeness (Lipinski’s rule of five) of the selected ligands [88]. The skin permeation coefficient was also predicted through this software. The SMILES of selected ligands were acquired from the PubChem database (https://pubchem.ncbi.nlm.nih.gov/) to predict these properties (accessed on 24 April 2025) [89].

#### 3.7.2. Molecular Docking Study

##### Target Protein Preparation

By reviewing the literature, the X-ray crystal structure of PPO3, a tyrosinase from *Agaricus bisporus* (PDB ID:2Y9X, resolution: 2.78 Å) along with the standard inhibitor tropolone were utilized [80]. The PDB file format of the protein was retrieved from the RCSB PDB (Protein Data Bank) database (https://www.rcsb.org/) (accessed on 22 April 2025) [90]. The hetero atoms were removed for preparation of the target protein. Furthermore, the kollman charges and hydrogen atoms were added to the target protein using AutoDockTools-1.5.7 to create a PDBQT file format of the protein.

##### Ligand Preparation

The SMILES of the shortlisted ligands were accessed from the PubChem database (https://pubchem.ncbi.nlm.nih.gov/) (accessed on 22 April 2025) [89]. The PDB file format of ligands was created using USCF Chimera 1.17.1 software [91]. These files were then converted into PDBQT file format using AutoDockTools-1.5.7 [92].

##### Molecular Docking

Molecular docking was performed using AutoDock Vina version 1.1.2 [93] where the protein file was kept rigid and the ligands were made flexible. The grid box dimensions were adjusted to 64 Å × 64 Å × 70 Å along the x, y, and z axis, respectively, to accommodate all the active sites, with spacing maintained at 1.0 Å. The grid box center coordinates were set at x = −7.400, y = −23.552, and z = −32.515. The number of default docking runs was set to 10 to obtain the best docked conformations. Kojic acid, a well-known standard inhibitor, was also docked with the protein for a comparison of interactions with other ligands. PyMOL was used to determine the best docked structures. On the basis of best binding affinity (ΔG) and conformations, 2D interactions between protein–ligand complexes were visualized through the BIOVIA Discovery Studio Visualizer (Discovery Studio Visualizer v.21.1.0.20298).

### 3.8. Molecular Dynamics Simulation (MDS)

MDS was performed to assess the conformational changes and stability of the best docked complexes using Gromacs 2024.2 software [94]. The topology files of the proteins and ligands were obtained using the SwissParam server [95]. The simulation process utilized the CHARMM27 all-atom force field [96]. The simulation box was generated and solvated using the TIP3P water model followed by neutralization with sodium and chloride ions. The system was equilibrated at a constant temperature of 300 K for over 100 picoseconds (ps) while using the constant volume (NVT) followed by the NPT ensemble where both pressure and temperature were maintained at 300 K with 1 bar pressure. The final production MD run was executed for 100 nanoseconds (ns) [97]. For trajectory file analysis, the coordinates were recorded at every 1 ps. The final generated trajectory files were used to evaluate the RMSD, RMSF, H- Bond, and Rg to analyze the stability of the protein–ligand complex [98].

### 3.9. Statistical Analysis

The results were expressed as mean ± standard deviation (SD) values of three independent replicates (*n* = 3). Statistical analyses for in vitro assays were performed using the One-way Analysis of Variance (ANOVA) followed by Dunnett’s multiple comparisons test using GraphPad Prism 8.0.1 software (GraphPad Software, San Diego, CA, USA). A *p* < 0.05 was considered statistically significant.

## 4. Conclusions

The present study revealed the successful establishment of *D. sissoo* callus cultures from a leaf explant and further subculture up to three stages. The antioxidant potential assay revealed that 20DC3 consists of potential antioxidants with a higher antioxidant potential compared with the parent explant and other calli. Moreover, further findings from the comparative analysis of phytochemical composition of all the methanolic extracts by GC⁃MS analysis revealed the presence of skin therapeutic phytochemicals at every stage of callus culture, which are reported in the literature for their numerous biological activities. Therefore, future investigations can be performed for the identification of these antioxidants, and their extraction optimization and further process development can be explored for commercial exploitation from each callus stage investigated. Additionally, in silico studies were also performed for exploring the inhibitory effects of phytochemicals against the tyrosinase enzyme for the prediction of probable drug candidates. The findings confirmed the possible role of the tested compounds in the inhibition of the enzyme. However, further in vitro and in vivo investigations are required in future studies to validate their possible role as anti-tyrosinase inhibitors. The present findings recommend the utilization of *D. sissoo* leaf callus cultures as a controlled and sustainable platform for the production of diverse phytochemicals with potential applications as skin therapeutics.

## Figures and Tables

**Table 1:** List of important phytochemicals detected in methanolic extracts of leaf explant (LE), 20th day callus (20DC), 1st subculture stage (20DC1), 2nd subculture stage (20DC2), and 3rd subculture stage (20DC3) with their molecular formula (MF), molecular weight (MW), comparative peak area %, and potential biological activities.

Compound Name	MF	MW (g/mol)	Peak Area %					Biological Activities
			LE	20DC	20DC1	20DC2	20DC3	
Chondrillasterol	C_29_H_48_O	412.7	1.57	4.00	4.16	2.57	-	Antimicrobial, Anti-inflammatory, Antioxidant [47,48]
Glyceryl palmitate	C_19_H_38_O_4_	330.5	-	-	-	1.77	-	Emulsifier, skin conditioning agent [49,50]
7,9-Di-tert-butyl-1-oxaspiro (4,5) deca-6,9-diene-2,8-dione	C_17_H_24_O_3_	276.4	1.05	3.21	3.17	3.51	2.53	Antineoplastic, Antimicrobial, Antiviral [51,52]
D-Ribose, 2-deoxy-bis (thioheptyl)-dithioacetal	C_19_H_40_O_3_S_2_	380.7	-	10.79	13.08	15.74	-	Antioxidant, Antimicrobial, Stimulate angiogenesis and wound healing [53]
Stearic acid	C_18_H_36_O_2_	284.5	-	0.82	-	-	-	Antifungal, Antitumor, Antibacterial, Anticancer [51,52]
Ethyl glucoside	C_8_H_16_O_6_	208.21	-	7.60	13.94	20.67	-	Antioxidant, antimicrobial, Anti-inflammatory [54,55]
Methyl palmitate	C_17_H_34_O_2_	270.5	-	0.48	0.40	0.65	-	Anti-Inflammatory, Antioxidant, Antitumor, immunostimulant, Antimicrobial [56,57,58]
Methyl α-d-galactopyranoside	C_7_H_14_O_6_	194.18	-	-	-	-	18.83	*
Isopropyl myristate	C_17_H_34_O_2_	270.5	-	2.15	1.76	4.64	2.26	Antioxidant, Antimicrobial, emollient. [51,52]
n-Hexadecanoic acid	C_16_H_32_O_2_	256.42	1.67	5.75	5.86	2.90	0.77	Anti-inflammatory, Antioxidant, hypocholesterolemic, nematicide, pesticide, anti-androgenic flavor, hemolytic [59,60,61]
1-Butyl-Cyclohexanol	C_10_H_20_O	156.26	0.16	0.36	-	-	0.42	Anti-inflammatory, Antioxidant [58]
4h-pyran-4-one, 2,3-dihydro-3,5-dihydroxy-6-methyl-	C_6_H_8_O_4_	144.12	-	8.15	6.23	7.19	-	Antioxidant, Antidiabetic, Antimicrobial, Anti-inflammatory [62,63]
Methyl linolenate	C_19_H_32_O_2_	292.5	0.39	0.55	0.37	0.59	0.17	Anti-inflammatory, Anticancer, Anti histaminic, Anti-melanogenesis Antioxidant [64,65]
Ethyl linoleate	C_20_H_36_O_2_	308.5	-	0.66	1.11	0.35	0.27	Anti-melanogenesis Antioxidant [65,66]
Phytol	C_20_H_40_O	296.5	7.22	1.09	1.14	2.13	0.92	Antimicrobial, Anticancer, Anti-inflammatory, Anti-diuretic, immunostimulatory, Antidiabetic [51,58,67]

* Activity not found in the literature. - Not detected.

**Table 2:** Drug likeness and ADME properties of the selected compounds detected in methanolic extracts of LE, 20DC, 20DC1, 20DC2, and 20DC3 identified by GC⁃MS analysis.

Compound Name	PubChem ID	Molecular Weight (<500)	Number of Rotatable Bonds	HBA	HBD	M Log *P*	Log *K_p_* (cm/s)	Number of Violations	Drug Likeness
Chondrillasterol	5283663	412.69	5	1	1	6.62	−2.92	1	Yes
Glyceryl palmitate (Monopalmitin)	14900	330.5	18	4	2	3.18	−3.82	0	Yes
7,9-Di-tert-butyl-1-oxaspiro(4,5)deca-6,9-diene-2,8-dione	545303	276.37	2	3	0	2.87	−5.28	0	Yes
D-Ribose, 2-deoxy-bis(thioheptyl)-dithioacetal	575898	380.65	18	3	3	3.36	−4.45	0	Yes
Stearic acid	5281	284.48	16	2	1	4.67	−2.19	1	Yes
Ethyl glucoside	91694274	208.21	3	6	4	−2.07	−9.19	0	Yes
Methyl palmitate	8181	270.45	15	2	0	4.44	−2.71	1	Yes
Methyl α-d-galactopyranoside	76935	194.18	2	6	4	−2.4	−9.37	0	Yes
Isopropyl myristate	8042	270.45	14	2	0	4.44	−2.83	1	Yes
n-Hexadecanoic acid	985	256.42	14	2	1	4.19	−2.77	1	Yes
1-Butyl-Cyclohexanol	138505	156.26	3	1	1	2.45	−5.15	0	Yes
4H-pyran-4-one, 2,3-dihydro-3,5-dihydroxy-6-methyl-	119838	144.12	0	4	2	−1.77	−7.44	0	Yes
Methyl linolenate	5319706	292.5	14	2	0	−1.77	−7.44	0	Yes
Ethyl linoleate	5282184	308.5	16	2	0	−1.77	−7.44	0	Yes
Phytol	5280435	296.5	13	1	1	5.25	−2.29	1	Yes

**Table 3:** Binding affinity (∆G) and molecular interactions of the best selected ligands against the target protein, tyrosinase enzyme.

Compound Name	Binding Affinity (kcal/mol)	No. of H-Bonds	Residue Involved In		
			H-Bond	Vander Waals	Pi-Alkyl Alkyl
Chondrillasterol	−8.7	0	-	Val276, Leu275, Ser282, Gly190, Asp191, Glu189, Phe188, Lys79, Thr187	Pro277, Pro284, Phe192, His285, Leu63, Tyr65
Glyceryl palmitate (Monopalmitin)	−7.5	0	-	Asn230, Gly223, Gly216, Ala215, Tyr201, Gly212, Asp269, Asn209, Met213	Trp227, Pro270, Ala220
7,9-Di-tert-butyl-1-oxaspiro(4,5)deca-6,9-diene-2,8-dione	−7.3	2	Lys379, Asp357	Glu356, Asp312, Gln307, Thr308	Tyr311, Trp358, Lys376
D-Ribose, 2-deoxy-bis(thioheptyl)-dithioacetal	−6.8	1	Asp30	Asn29, Ala122, Glu123, Gln126	Lys31, Ala127, Phe32
Stearic acid	−6.8	1	Ala323	Thr261, Asn260, Met257, His 85, Asn81, Glu322, Thr324, Ala80	Phe264, His244, Val283, Val248
Ethyl glucoside	−6.4	0	-	Thr308, Gln307, Tyr311, Asp312, Lys376, Glu356, Asp357, Lys379	Trp358
Methyl palmitate	−6.4	2	Asn81, Ala323	Ala80, Thr324, Glu322, Met257, Asn260, Thr261	Val283, Phe264, His244, Val248, His85
Methyl α-d-galactopyranoside	−6.3	2	Gln307, Gln307	Trp358, Lys379, Asp357, Glu356, Thr308, Tyr311, Asp312, Val313, Tyr314	-
Isopropyl myristate	−6.3	0	-	Tyr201, Gly216, Ala220, Thr261, Asn253, Asn230	Leu265, Pro270, Phe224, Trp227
n-Hexadecanoic acid	−6.1	1	Asn230	Tyr201, Gly216, Thr261, Ala252, Asn253	Leu265, Pro270, Phe224, Trp227, Ala220
Kojic acid *	−5.9	2	His85, His259	Ser282, Phe264, Gly281, Met280, Asn260, His61, Phe292, His296, His94	Ala286

* Standard inhibitor. - No Residues present.

**Table 4:** Representation of average values of Root Mean Square Deviation (RMSD) and Radius of Gyration (Rg) of the docked complexes of tested ligands with target protein (2Y9X) post molecular dynamics simulation.

Protein/Protein–Ligand Complex	Average RMSD (nm)	Average Rg (nm)
2Y9X	0.1591 ± 0.02	2.071 ± 0.01
2Y9X-Isopropyl myristate	0.1398 ± 0.02	2.063 ± 0.01
2Y9X-n-Hexadecanoic acid	0.1737 ± 0.02	2.071 ± 0.01
2Y9X-7,9-Di-tert-butyl-1-oxaspiro (4,5) deca-6,9-diene-2,8-dione	0.1567 ± 0.02	2.060 ± 0.01
2Y9X-Chondrillasterol	0.1523 ± 0.01	2.066 ± 0.01
2Y9X-D-Ribose, 2-deoxy-bis(thioheptyl)-dithioacetal	0.1771 ± 0.02	2.072 ± 0.01
2Y9X-Ethyl glucoside	0.1859 ± 0.03	2.078 ± 0.01
2Y9X-Glyceryl palmitate	0.1883 ± 0.03	2.076 ± 0.01
2Y9X-Methyl palmitate	0.1732 ± 0.02	2.073 ± 0.01
2Y9X-Methyl α-d-galactopyranoside	0.1726 ± 0.02	2.073 ± 0.01
2Y9X-Stearic acid	0.1626 ± 0.02	2.071 ± 0.01
2Y9X-Kojic acid (Standard)	0.1627 ± 0.02	2.069 ± 0.01

## Data Availability

The data is contained within this article and the Supplementary Materials, Figure S1, and Table S1.

## Supplementary Materials

The following supporting information can be downloaded at: https://www.mdpi.com/article/10.3390/molecules30173531/s1, Table S1: Values of IC_50_ (50% inhibition concentration) of all the extracts for DPPH scavenging activity. Figure S1. The GC⁃MS chromatograms of methanolic extracts of (a). leaf explant (LE), (b). 20th day callus (20DC), (c). 1st subculture stage (20DC1), (d). 2nd subculture stage (20DC2), and (e). 3rd subculture stage (20DC3).
