# Supplementary material for: Establishment of Callus Cultures from Dalbergia sissoo Leaf Explants for Production of Skin Therapeutics: An In Vitro and In Silico Study
Source: Molecules. 2025 Aug 29;30(17):3531. doi: 10.3390/molecules30173531 (PMC12430452; doi:10.3390/molecules30173531)
Supplement: Supplementary file 1 [file molecules-30-03531-s001.zip › molecules-3759556-supplementary.pdf]

Table S1: Values of IC_{50} (50% inhibition concentration) of all the extracts for DPPH scavenging activity

**Sample code**	**IC_{50} (μg/mL)**
LE	423 ± 4.82
20DC	649 ± 46.39
20DC1	726 ± 6.20
20DC2	749 ± 18.66
20DC3	273 ± 14.14

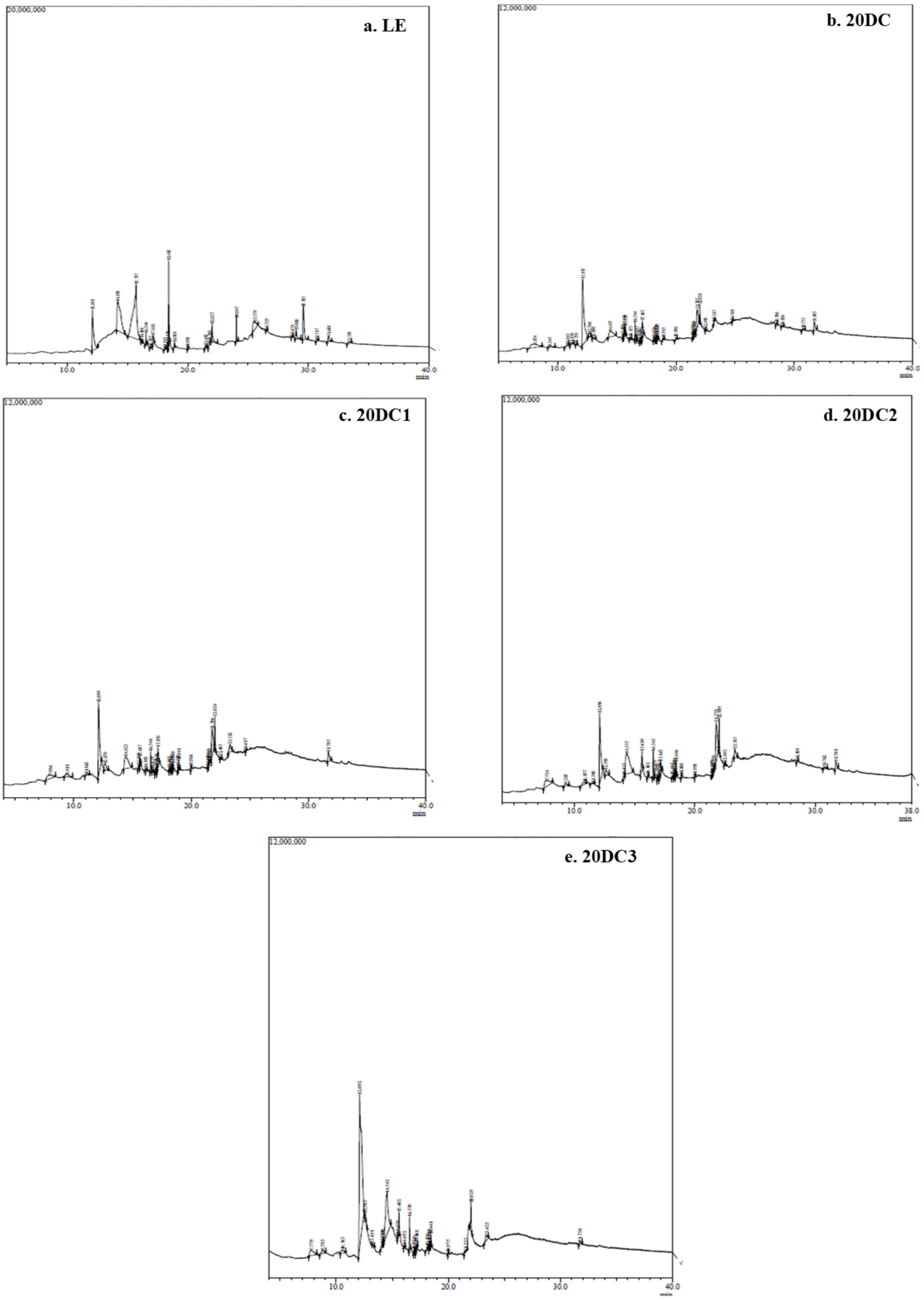

Figure S1. The GC-MS chromatograms of methanolic extracts of **(a).** leaf explant (LE), **(b).** 20th day callus (20DC), **(c).** 1st subculture stage (20DC1), **(d).** 2nd subculture stage (20DC2), and **(e).** 3rd subculture stage (20DC3).
